# More than redox, biological organic ligands control iron isotope fractionation in the riparian wetland

**DOI:** 10.1038/s41598-021-81494-z

**Published:** 2021-01-21

**Authors:** Elaheh Lotfi-Kalahroodi, Anne-Catherine Pierson-Wickmann, Olivier Rouxel, Rémi Marsac, Martine Bouhnik-Le Coz, Khalil Hanna, Mélanie Davranche

**Affiliations:** 1 Univ. Rennes, CNRS, Géosciences Rennes - UMR 6118, 35000 Rennes, France; 2 IFREMER, Unité de Géosciences Marines, 29280 Plouzané, France; 3 Univ. Rennes, Ecole Nationale Supérieure de Chimie de Rennes, CNRS ISCR UMR6226, 35000 Rennes, France

**Keywords:** Biogeochemistry, Environmental sciences

## Abstract

Although redox reactions are recognized to fractionate iron (Fe) isotopes, the dominant mechanisms controlling the Fe isotope fractionation and notably the role of organic matter (OM) are still debated. Here, we demonstrate how binding to organic ligands governs Fe isotope fractionation beyond that arising from redox reactions. The reductive biodissolution of soil Fe(III) enriched the solution in light Fe isotopes, whereas, with the extended reduction, the preferential binding of heavy Fe isotopes to large biological organic ligands enriched the solution in heavy Fe isotopes. Under oxic conditions, the aggregation/sedimentation of Fe(III) nano-oxides with OM resulted in an initial enrichment of the solution in light Fe isotopes. However, heavy Fe isotopes progressively dominate the solution composition in response to their binding with large biologically-derived organic ligands. Confronted with field data, these results demonstrate that Fe isotope systematics in wetlands are controlled by the OM flux, masking Fe isotope fractionation arising from redox reactions. This work sheds light on an overseen aspect of Fe isotopic fractionation and calls for a reevaluation of the parameters controlling the Fe isotopes fractionation to clarify the interpretation of the Fe isotopic signature.

## Introduction

Wetlands are recognized as key areas for controlling the fate of many inorganic and organic compounds in the environment. The capacity of wetlands to mobilize or retain them is mainly controlled by the redox processes that occur in response to water-level fluctuations^1–4^. Under these conditions, iron (Fe) and organic matter (OM) are two fundamental and interconnected chemical parameters. The Fe redox cycle and the subsequent species control the mobility of numerous trace elements and organic molecules^5–7^. In wetlands, when oxidizing conditions prevail, Fe is present as Fe(III) ions, clusters, and nanoparticles complexed to OM that directly controls the sizes of the Fe nanoparticles^8^. When reducing conditions prevail, Fe(II), solubilized by the reductive biodissolution (dissimilatory Fe reduction, DIR) of Fe(III) (nano)particles, is bound to OM solubilized in response to the rise in pH caused by the reduction reactions^2,6^. This binding limits Fe(II) adsorption or the precipitation of newly formed Fe(II)-bearing mineral, and increases the dissolution of Fe(III). What is less understood is how these complex interactions affect Fe isotopic signatures. Investigating Fe isotopic fractionation under such conditions will certainly provide invaluable information for fingerprinting elemental cycling processes and sources in wetlands.

Iron isotopic fractionation can result from both abiotic and biotic processes. Redox processes lead to large Fe isotopic fractionation ≈ 3‰ between the reduced and oxidized Fe species as predicted by density functional theory (DFT) calculations and single experimental conditions^9–13^. Under anoxic conditions, Fe(III)-oxyhydroxides are used as a terminal electron acceptor by DIR bacteria (e.g. *Geobacter*) resulting in the preferential release of light Fe isotopes into the solution compared to minerals^10,14^. Under oxic conditions, both partial abiotic and biotic oxidations/hydrolysis of aqueous Fe(II) produce an enrichment in heavy Fe isotopes of the particulate fraction up to 2.6‰^15–17^. We recently demonstrated that at equilibrium, the abiotic precipitation of dissolved Fe(III) as ferrihydrite does not significantly fractionate Fe isotopes^18^. However, kinetic fractionation of Fe isotopes was reported in several studies for partial and / or incomplete precipitation of Fe-containing minerals(e.g. siderite, hematite, mackinawite, pyrite and etc.) for specific conditions such as pH < 3^12^, high temperature (100 °C)^19^, biotic processes^20–22^, for oceanic amorphous Fe(III) oxides-silicon (Si) with high Fe(III) /Si ratio^23^. Although Skulan et al. observed kinetic Fe fractionation during rapid hematite precipitation^24^, they reported insignificant Fe fractionation for slow precipitation of Fe. However, the kinetic exchange of isotopes in presence of Fe(II) and Fe(III) is significant and faster than that in system containing only Fe(III). Biotic or abiotic oxidation processes are followed by the co-precipitation and/or aggregation of Fe(III)-oxyhydroxides with OM when it is present^8,25^. Under natural conditions, e.g. in some Arctic and sub-Arctic rivers, colloidal fractions enriched in OM and Fe exhibit a wide range of isotopic compositions (δ^56^Fe) from − 1.4 to + 2.8‰^26,27^. Furthermore, ligand-controlled dissolution of Fe oxides has been proposed to explain light Fe isotope enrichments in well-drained Cambisol and Podzols, while the OM-rich uppermost horizons of hydromorphic soils are enriched in heavy Fe isotopes^28,29^. Nevertheless, the underlying mechanisms by which Fe/OM complexation affects the Fe isotopic composition in the soil solution, are unclear.

To fill this knowledge gap, this study focuses on the impact of composition of OM sources and the potential Fe binding, on the overall Fe isotope composition of the soil solution under redox alternation. Experimental monitoring of a wetland soil solution undergone to anoxic and oxic periods was used as a means to emulate natural redox cycles. Our main objectives were (1) to investigate the evolution of the Fe isotopic composition of the soil solution in response to 3 successive redox cycles, (2) to highlight the impact of OM on the mechanisms responsible of the Fe isotopic signature, and (3) to compare and validate our experimental findings with natural field data.

## Results and discussion

### The soil and redox oscillations system

**Figure 1:**
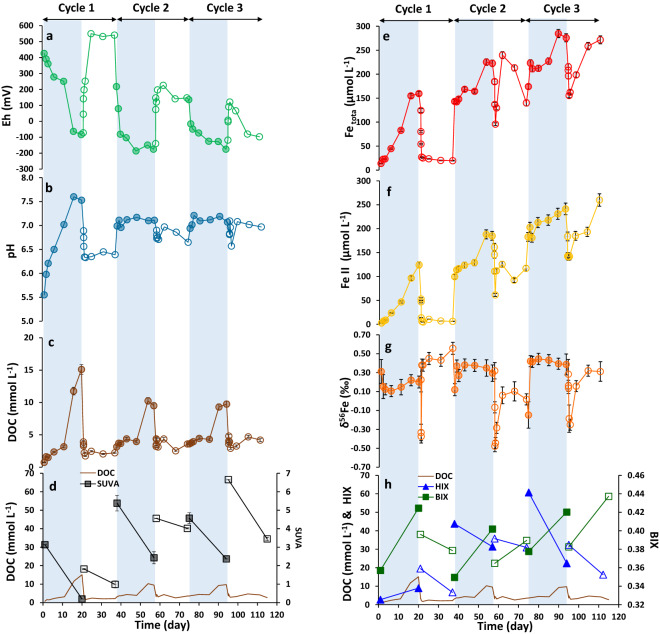
Evolution of Eh (**a**), pH (**b**), DOC (**c**), SUVA (**d**), Fe_tot_ (**e**), Fe(II) (**f**), δ^56^Fe (**g**) and the BIX and HIX indexes (**h**) versus time (day) during the anoxic/oxic cycles. Eh and pH were measured in the soil suspension and other parameters were determined in filtrates of 0.2 µm. The blue zones illustrate the anoxic periods and the white ones the oxic periods. The filled and empty symbols correspond to samples under anoxic and oxic conditions, respectively. Analytical error bars were calculated by (i) measuring the standard for DOC, Fe(II) Fe_tot_, and δ^56^Fe (2SD) analyses, (ii) average of Replicates analyses (n = 2 or 3) for SUVA, and (iii) Eq. (3) if there were replicates of analysis.

A soil sample corresponding to an organo-mineral horizon (Ah) of a Planosol (WRB classification) was collected in a riparian wetland in the Kervidy-Naizin sub-catchment (Brittany, France, Materials and Methods). Redox oscillations, as observed in the riparian wetland, were experimentally simulated using soil incubations under successive anoxic (in an anaerobic chamber) and oxic conditions (ambient conditions) at room temperature. The concentrations of Fe_tot_, Fe(II), dissolved organic carbon (DOC), Fe isotopic composition (δ^56^Fe), pH, and Eh were measured at regular time intervals (Fig. 1). Dissolved organic carbon (DOC) was characterized by three-dimensional (3D) fluorescence. Three indexes, specific UV absorbance (SUVA), humification index (HIX), and biological index (BIX) were calculated to characterize OM. Increasing in SUVA, HIX, and BIX indicates an increase in the aromaticity, humic character, and autochthonous biological origin of the OM sources, respectively^30–32^. Finally, a series of filtrations at 3 µm and 0.2 µm and ultrafiltration at 30 kDa were performed on the soil suspension at the end of the anoxic/oxic periods 1 and 3.

During anoxic periods, pH increased in response to the consumption of H^+^ by the reduction reactions (Fig. 1a,b), and Fe(II) was solubilized through the reductive dissolution of the soil Fe-containing minerals and aggregates (e.g. Fe(III) oxyhydroxides). This process is driven by the activity of heterotrophic bacterial community using soil OM as C source, as previously demonstrated by Dia et al.^33^ for the same soil sample (Fig. 1c,e,f; Supplementary Table S1). Our experiments showed that a proportion of Fe in the 0.2 µm–30 kDa fraction occurred as Fe(III) (e.g., Fe-containing silicates and Fe-OM) compounds (Table 1). Although some Fe(II) was bound to particulate and colloidal OM in the > 30 kDa fractions, Fe(II) mainly occurred as soluble complexes in the < 30 kDa fraction as previously demonstrated by Davranche et al.^6^. The solution was systematically under-saturated with respect to ferric and ferrous oxides. Speciation calculations, using the PHREEQC-MODEL VI^34^ showed that at the end of each anoxic period, Fe(II) was complexed at 100%, 96% and 93% with OM for anoxic period 1, 2 and 3 respectively,. Dissolved organic carbon was released via desorption from soil minerals due to the pH rise, and was also produced by bacterial metabolic activities^2,3,7,33^. These results were supported by the SUVA, HIX and BIX variations indicating production of aromatic OM at the beginning of each anoxic period (i.e. aromatic OM desorption), whereas biological and less aromatic OM dominated (i.e. bacterial metabolite activity) at the end of each anoxic period (Fig. 1d,h; Supplementary Table S2).

**Table 1:** Chemical and isotopic compositions of the filtrated and ultrafiltrated samples for anoxic and oxic periods 1 and 3.

Size fraction	DOC (mmol L^−1^)	Fe(II) (µmol L^−1^)	Fe_tot_ (µmol L^−1^)	Fe(II)/Fe_tot_	Fe_tot_/Fe _soil_ (%)	δ^56^Fe ± 2SD(‰)
*Anoxic 1*						
> 3 µm	nd	nd	6746.4 ± 365.3	nd	96.7	0.43 ± 0.09
3–0.2 µm	2.2 ± 1.1	22.1 ± 9.6	68.5 ± 8.3	0.32	1.0	0.75 ± 0.27
0.2 µm–30 kDa	7.0 ± 0.8	54.9 ± 7.1	88.6 ± 5.2	0.62	1.3	0.83 ± 0.14
< 30 kDa	7.7 ± 0.4	69.2 ± 3.5	70.9 ± 2.1	0.98	1.0	− 0.59 ± 0.09
*Anoxic 3*						
> 3 µm	nd	nd	7518.9 ± 389.8	nd	95.8	0.43 ± 0.14
3–0.2 µm	0.0 ± 0.1	28.3 ± 18.1	54.7 ± 13.2	0.52	0.7	ns
0.2 µm–30 kDa	2.4 ± 0.6	170.2 ± 12.6	205.8 ± 8.7	0.83	2.6	0.72 ± 0.16
< 30 kDa	7.4 ± 0.4	71.1 ± 3.6	70.1 ± 2.1	1.00	0.9	− 0.59 ± 0.10
*Oxic 1*						
> 3 µm	nd	nd	7784.1 ± 399.8	nd	99.2	0.50 ± 0.09
3–0.2 µm	1.3 ± 0.2	3.2 ± 0.6	44.9 ± 2.0	0.07	0.6	0.37 ± 0.10
0.2 µm–30 kDa	0.4 ± 0.1	6.0 ± 0.3	5.7 ± 0.7	1.00	0.1	0.40 ± 0.25
< 30 kDa	1.8 ± 0.1	< LOD	13.9 ± 0.4	nd	0.2	0.62 ± 0.05
*Oxic 3*						
> 3 µm	nd	nd	7807.1 ± 389.6	nd	99.5	0.56 ± 0.13
3–0.2 µm	0.3 ± 0.2	12.1 ± 0.7	20.3 ± 1.0	0.60	0.3	0.45 ± 0.11
0.2 µm–30 kDa	1.2 ± 0.2	1.3 ± 0.1	3.3 ± 0.4	0.40	0.1	ns
< 30 kDa	1.5 ± 0.1	< LOD	8.2 ± 0.2	nd	< 0.1	0.59 ± 0.14

*DOC* dissolved organic carbon, *ns* not significant, *nd* not determined, < *LOD* below the limit of detection.

During oxic periods, the pH slightly decreased in response to OH^−^ consumption by the Fe(III) hydrolysis^3,4^ (Fig. 1b; Supplementary Table S1). The concomitant decrease in Fe(II) and Fe_tot_ can be explained by Fe(II) oxidation/precipitation, as Fe(III) aggregates, clusters and nano-oxides^8,25^ (Fig. 1e,f; Supplementary Table S1). The presence of Fe nano-aggregates was confirmed by electron transmission microscopy (TEM) observations (Supplementary Fig. S1). Iron(II) was rapidly oxidized, with about 95%, 67%, and 42% of Fe(II) being oxidized within 10 h for the oxic periods 1, 2, and 3, respectively. Moreover, during the first hour of the three oxic periods, Fe oxidation rates reached 10, 3, and 8 mmol L^−1^ d^−1^, respectively. Then, these rates dropped to 0.3, 0.003, and 0.001 mmol L^−1^ d^−1^ for the second hour of oxidation, respectively. These results are within the Fe oxidation rate range (0.4–73 mmol L^−1^ d^−1^ ) obtained in natural groundwater settings, in the presence of dead and living microorganisms^35^. The rapid and extensive Fe(II) oxidation suggests that homogenous autocatalytic (dissolved Fe(II) and O_2_) and/or heterogeneous abiotic oxidation (accelerated by precipitated Fe nano-oxides) occurred in our system^36,37^. The separation of the different Fe phases using filtration/ultrafiltration techniques confirmed that Fe was mainly oxidized and precipitated in the > 0.2 µm particulate fraction (Table 1). The DOC concentration decreased in response to its aggregation with the newly formed Fe(III) nano-oxides^8,25^ (Fig. 1c). SUVA and HIX were lower at the end of the anoxic periods than at the beginning of the following oxic periods, indicating that the biological and less aromatic organic molecules were eliminated from the soil solution by switching to oxic conditions (Fig. 1d,h; Supplementary Table S2). The rate of these decreases cannot be explained by their biological degradation (mineralization) but rather by their aggregation/precipitation with the Fe(III) species. Another interesting point is that Fe(II) was maintained in the solution even under oxic conditions during the last two oxic periods. Catrouillet et al.^34^ and Rose and Waite^38^ demonstrated that Fe(II) form strong bidentate complexes with the OM carboxylic functional groups which strongly influence its solubility. Speciation calculation using PHREEQC-MODEL VI showed that,in solution, Fe(II) was bound at 100%, 95% and 64% with dissolved OM at the end of oxic periods 1, 2, 3, respectively. Iron(II)-OM binding also prevents possible adsorption of Fe(II) onto the newly formed Fe-oxides. Moreover, the Fe(II) complexation with OM limits their oxidation/hydrolysis^38,39^. The OM characterization (SUVA, HIX, BIX) and Fe(II) concentration thus showed that some Fe(II) was maintained in the soil solution by its complexation to the more aromatic dissolved organic molecules. Finally, during the two oxic periods 2 and 3, Fe(II) and Fe_tot_ increased in the soil solution over time, suggesting that Fe previously precipitated as aggregated at the beginning of the oxidation, were progressively released into the solution (Fig. 1e). This increase was concomitant with the increase in DOC and BIX as well as with the SUVA decrease suggesting that Fe was solubilized as Fe-organic complexes, with mostly fresh biological organic compounds. The evolutions of DOC, BIX, and SUVA also support the fact that the bacterial metabolic activities increased over time under these oxic periods.

The general variation of some parameters over time suggests that no steady-state has been reached: (1) the amplitude of pH variations became smaller, probably due to increase in DOC concentration acting as a buffer^4,7^; (2) Eh globally decreased, suggesting that the establishment of oxidizing conditions is more difficult to achieve during successive redox cycles, whereas (3) DOC concentration decreased during the anoxic periods and increased during the oxic periods, and (4) finally, the Fe(II) and Fe_tot_ concentrations increased continuously (Fig. 1). This increase can be explained by the increasing reductive biodissolution of Fe(III) and the increasing Fe binding with the increasing amount of dissolved organic ligands in the solution during the anoxic periods (regardless of the sources of DOC), as previously reported^4^. This increase is also supported by the remaining Fe(II) under oxic periods.

### Mechanisms governing iron isotopic fractionation

The δ^56^Fe of the total dissolved Fe evolved between the anoxic and oxic periods, with larger fluctuations during the oxic periods. More generally, the δ^56^Fe of the anoxic periods was higher than that of the oxic periods except for the oxic period 1 which exhibited the highest δ^56^Fe with values close to that of the initial wetland soil at 0.43 ± 0.08‰ (Supplementary Table S1).

The anoxic period 1 displayed an initial decrease in δ^56^Fe at 0.10 ± 0.06‰, followed by a rebound. As the reduction progressed with increasing of Fe(II), δ^56^Fe remained constant within uncertainty until day 11, at which time the heavy Fe isotopes progressively increased in solution. (Fig. 1f,g; Supplementary Table S1). Previous studies demonstrated that dissimilatory Fe reduction (DIR) can produce millimolar quantities of light Fe isotopes, with isotopic fractionation varying from − 1.5 to − 0.5‰^10,11,14,40^. The limited δ^56^Fe′ (− 0.32‰ maximum) could be explained by several hypotheses: (1) the Fe reduction is almost quantitative which is unlikely since only 3% of the soil total Fe was dissolved; although 23% of soil total Fe was estimated to be reducible in this soil sample^41^; (2) solubilized Fe(III) was from heavier pool than bulk soil. Based on a mass balance calculation, we have calculated that this source should vary within a range from − 0.7 to − 0.2‰, with Δ^56^Fe_Fe(II)aq-mineral_ variations from − 1.5 to − 0.5‰^10,11,14,40^. This is also unlikely because isotopic signature of solubilized Fe(III) was not heavier than that of bulk soil (0.43 ± 0.08‰). Moreover, data from literature show that δ^56^Fe of the reducible Fe in soils (e.g. cambisol and gleysol) and marine sediments vary from − 0.15 to 0.20‰^42–44^; (3) the δ^56^Fe of the soil solution corresponded to a mixture of the contrasted isotopic Fe signature released from the soil by a mechanism other than DIR, for example the release of small amount of Fe(III) colloids with heavy isotopic signature^45,46^. This hypothesis is also unlikely since, in this soil, DIR is the driver of the Fe(II) production in response to soil water-saturation^2,7,33,47^, (4) finally, Chanda et al.^48^ suggested that Fe(II) adsorption onto ferrihydrite aggregates results in an equilibrium fractionation at 2.36 ± 0.26 ‰ between the Fe(II) solution and the aggregates. However, such fractionation is also unlikely because the experiment of Chanda et al.^48^ differs strongly from ours, especially with respect to Fe/OC ratio and because the δ^56^Fe value of the present soil solution was too low to be produced by such processes (which is strongly limited by the Fe(II) binding by OM). Therefore, none of the hypotheses can support the obtained limited δ^56^Fe′_solution-soil_, and a fourth process independent of the Fe source should thus be considered.

Following a time lag marked by the release of aromatic DOC (high SUVA and HIX) desorbed from the soil mineral and a weak Fe(III) reduction rate of 0.2 µmol L^−1^ h^−1^, the bacterial community grew and released a high amount of biological organic molecules (high BIX, low SUVA) and reduced Fe at higher rate, 0.4 µmol L^−1^ h^−1^ (Fig. 1c,d, 1e–h; Supplementary Tables S1 and S2). This bacterial growth was surprisingly concomitant with a release of heavy Fe isotopes into the solution (δ^56^Fe increased from 0.10 ± 0.06‰ to 0.20 ± 0.06‰). For the subsequent anoxic periods 2 and 3, the bioreduction of Fe(III) to Fe(II) produced an initial enrichment in light Fe isotopes as compared to the soil (δ^56^Fe′_solution-soil_ at − 0.31 ± 0.10‰ and − 0.58 ± 0.16‰, respectively). As the reduction progressed, the δ^56^Fe of both soil solutions increased progressively from 0.12 ± 0.06‰ to 0.29 ± 0.09‰ and − 0.15 ± 0.06‰ to 0.39 ± 0.11‰ for the anoxic periods 2 and 3, respectively (Fig. 1g; Supplementary Table S1). This enhancement was concomitant with a large increase in bacterial OM indicated by both high BIX and low SUVA (Fig. 1c,d and g,h). In both anoxic periods 1 and 3, the 0.2 µm–30 kDa colloidal fractions had the highest δ^56^Fe (0.83 ± 0.14‰ and 0.72 ± 0.16‰) while the soluble < 30 kDa fractions exhibited negative δ^56^Fe (− 0.59 ± 0.09‰ and − 0.59 ± 0.10‰, respectively). Such isotopically heavy Fe pool in the > 30 kDa fractions reflected the release of isotopically light Fe in the < 30 kDa fractions (Table 1). Moreover, the results showed that in the 0.2 µm–30 kDa fractions, Fe(II) bound to organic colloids, is enriched in heavy Fe isotopes by 1.33 ± 0.28‰ and 1.10 ± 0.71‰ (Table 2; Supplementary Table S3), respectively, as compared to the < 30 kDa soluble fractions where Fe(II) is bound to small soluble organic molecules (Supplementary Figs. S2 and S3). It is important to note here, that all of the OM fluorescence indexes (SUVA, BIX, and HIX) showed that this OM was dominated by biological OM (Supplementary Table S2). All these results suggest that the large fractions were composed of large biological organic by-products, which were consistent with the molecular weight of biological exopolysaccharides with size range from 5700 to 80 kDa, approximatively^49,50^. Therefore, under reducing conditions, the large biological molecules (or colloids) of OM bound the heavy Fe isotopes and kept them in the soil solution preventing their adsorption on the soil components. Furthermore, the evolution of the δ^56^Fe indicated that at the beginning of each anoxic period, the bacterial reduction of the Fe(III)-oxides effectively occurred but that, in a second step, the heavy Fe isotopes are preferentially bound by large biological-derived OM.

**Table 2:** Iron isotopic fractionation (Δ^56^Fe_A–B_) between the size fraction A (on the left, yellow column) and the size fraction B (on the top, blue row). The values were calculated by subtracting the δ^56^Fe of the fraction from the δ^56^Fe of the fraction A.

Δ^56^Fe_A–B_	Size fraction B		
Size fraction A	3–0.2 µm	0.2 µm–30 kDa	< 30 kDa
*Anoxic 1*			
> 3 µm	− 0.32 ± 0.28	− 0.40 ± 0.17	1.01 ± 0.13
3–0.2 µm	–	ns	1.33 ± 0.28
0.2 µm–30 kDa	ns	–	1.41 ± 0.17
*Anoxic 3*			
> 3 µm	ns	− 0.29 ± 0.21	1.02 ± 0.17
3–0.2 µm	–	ns	1.10 ± 0.71
0.2 µm–30 kDa	ns	–	1.31 ± 0.19
*Oxic 1*			
> 3 µm	0.13 ± 0.13	ns	− 0.12 ± 0.10
3–0.2 µm	–	ns	− 0.25 ± 0.11
0.2 µm–30 kDa	ns	–	ns

The Δ^56^Fe_A–B_ errors were calculated using Eq. (7). For the oxic period 3, after vent removing, no significant Fe isotopic fractionation was observed, because the high uncertainty obtained from error propagation calculation (Supplementary Table S3).

– Not defined isotopic fractionation, *ns* no significant error propagation.

The oxic periods were initially marked by a sharp drop of δ^56^Fe of the soil solution followed by a rebound to heavier values. The highest δ^56^Fe values were observed at the end of the oxic period 1 (Fig. 1g; Supplementary Table S1). For the oxic period 1, δ^56^Fe dropped from 0.23 ± 0.13‰ to − 0.37 ± 0.07‰ over the first 2 h (Fig. 1g). This large drop was concomitant to a large decrease in the Fe(II), Fe_tot_, and DOC concentrations compared to their concentrations at the end of the previous anoxic period (Fig. 1c,e,f). All these decreases were driven by the Fe(II) oxidation/hydrolysis as Fe(III) clusters and nano-oxides and their subsequent aggregation/sedimentation with OM^8,25^. The decrease in Fe_tot_ corresponded to oxidation/precipitation of 66% of the Fe produced in the anoxic period 1. Previous works demonstrated that because of the isotopic exchange kinetic between Fe(III) and Fe(II), both abiotic and biotic Fe(II) oxidation enriched the Fe(III)-rich precipitates in heavy Fe isotopes^12,51,52^. The large oxidation rate of Fe(II) cannot be mediated by bacteria as previously discussed. Therefore, in this experiment, the light Fe enrichment was not produced by bacterial oxidation but rather by abiotic mechanisms. Using Rayleigh fractionation model with an Fe isotope fractionation at 2.9‰^12^ for biotic Fe(II) oxidation to Fe(III), we determined that an extent of 66% of Fe(II) oxidation would translate a drop of − 2.8‰ for the initial Fe(II) pool (Supplementary Fig. S4). This large shift, however, was not fully expressed during the oxic period because the dissolved Fe pool is also affected by the formation of isotopically heavy Fe(III) clusters and nano-oxides embedded in the organic matrix that further aggregated and sedimented. This process was accompanied by an increase in SUVA and HIX, as well as a decrease in BIX, confirming the disappearance of the larger biological and less aromatic OM from the soil solution and therefore their aggregation/sedimentation with Fe(III) (Supplementary Table S2). Therefore, the Fe isotope composition of the soil solution was driven by the Fe binding to large biological organic by-products and their concomitant aggregation/sedimentation.

Following the initial δ^56^Fe drop due to partial Fe(II) oxidation, δ^56^Fe increased rapidly to 0.38 ± 0.06‰ and finally reached 0.56 ± 0.06‰ at the end of the oxic period 1. The variations in the enrichment of the Fe isotopes compared to the soil, δ^56^Fe′_solution-soil_, were insignificant for this second step (Supplementary Table S1). A slight increase was observed between 22 and 37 days for the DOC and Fe(II) concentrations in the second step. Moreover, the decreasing SUVA and fluorescence indexes indicated that more biological OM was released in the soil solution probably in response to the biological by-product degradation, anoxic bacterial cell lysis, and/or aerobic bacterial metabolic activities resumption. The filtration/ultrafiltration experiments showed that the < 30 kDa fraction had the heaviest δ^56^Fe at 0.62 ± 0.05‰ (Table 1). The calculated data showed 71% of the < 0.2 µm fraction consisted of the < 30 kDa fraction (Table 1). With regard to the SUVA and fluorescence indexes, OM composing the < 30 kDa fraction was probably derived from the degradation of larger biological-derived OM that had preferentially bound heavy Fe isotopes, during the previous anoxic period. As oxidizing conditions progressed, heavy Fe isotopes were released into the soil solution and gradually controlled the δ^56^Fe.

Both the following oxic periods presented the same δ^56^Fe evolution that can be explained by the same mechanisms, with a slowdown in the δ^56^Fe increase. For oxic periods 2 and 3, the δ^56^Fe trend showed a positive direction after 10 and 24 h, respectively, in response to more difficult oxidation of Fe(II) with the increase in redox cycles numbers^4^. These results showed that more and more Fe(II) remained in the soil solution over the oxic periods. In the oxic periods 2 and 3, the oxidation was not therefore quantitative and resulted in a lower δ^56^Fe than that obtained in the oxic period 1.

### The implication for seasonal redox processes in wetland soils

**Figure 2:**
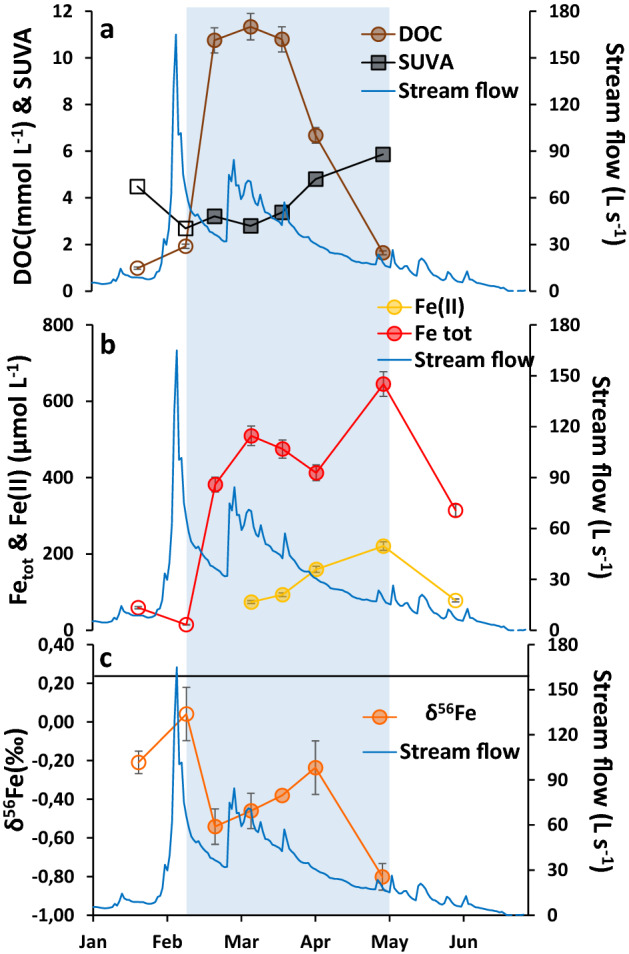
Evolution of DOC and SUVA (**a**), Fe(II) and Fe_tot_ (**b**), and δ^56^Fe (**c**) in 2017 versus date during the seasonal anoxic/oxic periods. The stream discharge is illustrated in blue. The straight line (**c**) demonstrates the Fe isotope composition of the wetland soil at 0.26 ± 0.07‰ in the sampled soil solution horizon (the chemical composition of the soil was determined by SARM, Supplementary Table S5). The blue zone illustrates the anoxic periods. The filled and empty symbols correspond to samples under anoxic and oxic conditions, respectively. Analytical error bars were calculated by (i) measuring the standard for DOC, Fe(II) Fe_tot_, and δ^56^Fe (2SD) analyses, and (ii) Eq. (3) if there were replicates of analysis.

Our hypothesis was confirmed by the field dataset collected from the Mercy wetland (Brittany, France) during the seasonal redox cycle in 2017. Figure 2 illustrates the evolution of the DOC concentration and SUVA (Fig. 2a), Fe(II) and Fe_tot_ concentrations (Fig. 2b) and Fe isotopic composition (δ^56^Fe) (Fig. 2c; Supplementary Table S4) of the soil solution samples (< 0.2 µm) collected from the wetland soil. From January to mid-February, the water level was low and the oxidizing condition was set up with low Fe_tot_ and DOC concentrations (Fig. 2a,b). The high SUVA value indicated the presence of aromatic OM. The δ^56^Fe of the soil solution was equal to − 0.21 ± 0.06‰ versus 0.26 ± 0.07‰ for the soil (at the depth of sampling; Supplementary Table S4). With the increase in seasonal rainfall, low-aromatic OM was solubilized as shown by the SUVA decrease and DOC increase. The consequence was an increase in δ^56^Fe (0.04 ± 0.14‰) of the soil solution. Presumably, Fe binding to moderately aromatic OM (SUVA = 2.7)^53^ or to an average of the aromatic and biological OM, enriched the soil solution in heavy Fe isotopes.

With rainfall increase, the soil was progressively waterlogged, allowing reducing condition to occur as shown by the increases in the Fe(II) and DOC concentrations. The production of Fe(II) was accompanied by a large decrease in δ^56^Fe at − 0.54 ± 0.09‰. This large difference of − 0.58 ± 0.09‰ between the oxidizing and reducing conditions is consistent with a biological mediated reduction of the soil Fe(III). Then, δ^56^Fe increased from − 0.54 ± 0.14‰ in mid-February to − 0.24 ± 0.14‰ in early April. Until mid-march, the increase corresponded to the highest DOC concentration and lower SUVA (from 3.2 to 3.4). These organic molecules complexed preferentially to heavy Fe isotopes promoting their retention in the solution, in agreement with our experimental hypothesis. In early April, DOC decreased while Fe(II) and SUVA were still increasing. Dissolved organic carbon was thus disconnected from the redox processes and instead decreased in response to the water level drop whereas reducing processes were still occurring (Fig. 2a). The SUVA values increased in response to the preferential leaching of the most labile OM. As a consequence, δ^56^Fe increased to − 0.24 ± 0.14‰ showing an increase in the amount of heavy Fe isotopes in the soil solution by the remaining more aromatic OM. Finally, in May, DOC was largely leached (loss of 82% in mid-March) and the remaining OM had a high SUVA, suggesting the presence of aromatic organic compounds in the soil solution. However, the Fe reduction was still on-going, as highlighted by the highest Fe(II), and δ^56^Fe decreased to − 0.80 ± 0.07‰. The molar DOC/Fe_tot_ ratio varied from 3 to 131 during the flooding soil period and more precisely from 28 to 3 between February and May after soil leaching, with a very low DOC concentration of 1.6 mmol L^−1^ in May. Both the very low DOC concentration and the decrease in the DOC/Fe_tot_ ratio provided evidence that there was not enough OM to control and dominate the Fe isotopic signature. Therefore, this low δ^56^Fe is explained by the bacteria-mediated reduction process^10,11,40^ (Fig. 2c). As a consequence, the soil solution is enriched in light Fe isotopes subsequently to the lowering of the water level.

The field data demonstrated that the preferential binding of heavy Fe isotopes to moderately aromatic organic ligands is a key process of the Fe isotopic signature during the redox cycle. However, they also demonstrated the importance of the water flow in open systems, which controls the DOC flux.

## Conclusion

During an anoxic period, DIR fractionates the Fe isotopes to enrich the soil solution with isotopically light Fe(II). But with development of reducing condition, the released Fe(II) is complexed by dissolved OM, which favors the binding of heavier Fe isotopes. The combined results of these two processes thus lead to a soil solution with Fe isotopes compositions, contrary to previous experimental studies^10,11,40^. Under reducing conditions, δ^56^Fe cannot therefore be a diagnostic of Fe reduction. Here, we demonstrated that Fe isotope fractionation during microbial Fe(II) production under natural conditions is masked by the binding of isotopically heavy Fe(II) with fresh biological-derived OM. While ephemeral, partial Fe(II) oxidation combined to the aggregation/sedimentation of Fe(III)-OM associations upon rapid oxygenation of soils, is a key factor to produce the most negative Fe isotope fractionation. The Fe isotopic compositions themselves are thus unable to discriminate between DIR and abiotic Fe oxidation pathways, but the extent of Fe isotope fractionations between bulk soil, soluble, and Fe-OM colloids are a consequence of the contrasted reactivity (i.e. turn-over rate) of these inorganic and organic Fe pools.

Even though redox variations remain the major controlling process of the Fe cycle, organic ligands should not be underestimated. The present study shed new light on the interpretation of Fe isotopic results in field data, in particular for arctic streams characterized by large Fe isotope fractionation among organic-rich colloidal and particulate pools^26,27^. Such large variations are likely the expression of successive oxic and anoxic periods associated with seasonal permafrost thawing. Similar effects are expected in soil solutions, in response to soil water saturation/desaturation cycles and their subsequent redox variations. For steady-state conditions, Fe isotope compositions are controlled by Fe-OM interactions, resulting in heavy Fe isotope signatures in soil solution whereas oxic/anoxic variations producing the light signatures. Therefore, the interactions between both abiotic and biotic processes must be unraveled to better interpret Fe isotopic composition in soil and paleosol environments.

## Materials and methods

### Site description and soil collection

One kg of soil was collected in February 2018 in the uppermost organo-mineral horizon of the Mercy wetland of Kervidy-Naizin located in Brittany in western France at 48°00′42.4″ N and − 2°50′20.2″ E (decimal degrees). This sub-catchment has been monitored since 1991 (OZCAR Observatory) and fully investigated in the study of colloid mobilization and its control on contaminants (e.g. arsenic)^6,47,54,55^. The hydrological, pedological, and geochemical contexts are therefore well known and well documented^1,56–58^. This soil is mainly dominated by clay (42%), quartz (30%), and Fe(III)-oxyhydroxides (3.5%). The clay fraction is composed of kaolinite, smectite, mica, hydroxyl-aluminous, vermiculite and interstratified minerals^59^. Geochemical compositions of the soil was determined by the Service d'Analyse des Roches et des Minéraux (SARM, France, Supplementary Table S5). The soil was sieved at 2 mm using a nylon sieve and stored in the dark at 4 °C to minimize the latent biological activity. The initial soil moisture was measured at 20.5% (wt).

### Experimental setup

To avoid contamination, all the reactors and containers were trace-metal pre-cleaned with 10% (v/v) HNO_3_ for 24 h at 45 °C, rinsed with ultrapure 18 MΩ water (Milli-Q system, MILLIPORE) for 24 h at 45 °C, and dried at 30 °C. Soil solution anoxic (20d)/oxic (17d) incubation consisted of a series of six reactors alternatively placed under either inert (N_2_) atmosphere using an anaerobic chamber (JACOMEX, O_2_ < 5 ppm) during the anoxic period or under ambient atmosphere during the oxic period. A reactor was sacrificed at the end of each period. Oxic conditions were slowly established using a rubber stoppers pierced with a 0.3 mm syringe needle in order to prevent rapid Fe oxidation. The soil/solution mass ratio was 1:20 (wt., considering soil moisture) for a 2L total volume. The solution was prepared with 0.06 µmol L^−1^ of NaNO_3_, 0.14 µmol L^−1^ of NaCl, and 0.1 mmol L^−1^ of Na_2_SO_4_. The six reactors were continuously stirred at 20 °C, in the dark, allowing three anoxic–oxic cycles to be followed over 111 days. The samples were collected relative to time. The pH and Eh of the soil suspension were monitored before sampling. The sampling was performed at 1 min; 2, 3, 6, 11, 16, and 20 days for the anoxic periods, and at 1 min, 1, 2, 10 h and , 1, 4, 10, and 16 days for the oxic periods .Roughly 20 mL of the soil suspension was collected, and filtered at 0.2 µm with a cellulose acetate membrane (SARTORIUS). At the end of each period except the second cycle (anoxic and oxic period 2), approximately 150 mL of the soil suspension was collected and subsequently filtered at 3 µm and 0.2 µm with cellulose acetate filters (SARTORIUS, details in Supplementary file). Roughly 15 mL of the < 0.2 µm fraction was ultrafiltered at 30 kDa (Vivaspin, SARTORIUS). Filtration/ ultrafiltration was performed at 3 µm, 0.2 µm, and 30 kDa to separate (1) fresh biological OM containing fragment and leaf debris in which Fe is found as old inherited Fe(III) oxyhydroxides (e.g. goethite) or nano-particles of Fe(III) embedded within the OM matrix (3–0.2 µm) from (2) colloidal fraction (0.2 µm–30 kDa), where OM occurred as aggregates of humic substances embedding Fe(III) nano-particles and clusters, and finally to separate (3) small Fe monomers or oligomers bound to OM molecules (< 30 kDa). All the filtrations and ultrafiltration were performed under anoxic conditions for the anoxic period. The Fe isotopic composition, total Fe dissolved (Fe_tot_) and Fe(II) concentrations and dissolved organic carbon (DOC) was determined for each sample. The OM was characterized by three-dimensional (3D) fluorescence at the beginning and the end of each period for the < 0.2 µm fractions. Three indexes, the specific UV absorbance (SUVA), the humification index (HIX), and the biological index (BIX), were calculated. At the end of oxic period 3, the rubber stopper of the reactor was completely removed. When Fe(II) was no longer measurable, the soil suspension was sampled and analyzed according to the protocol described above. The procedural blank was assessed by processing ultrapure water throughout the entire filtration and ultrafiltration steps. All of the filters were previously washed with ultrapure water. The ultrafiltration cells were cleaned using 0.1 mol L^−1^ of NaOH and ultrapure water.

### Field soil solution sampling

Samples of wetland soil waters were collected in 2017 using soil solution collectors emplaced in the Mercy wetland at a depth of 55 cm. These soil waters correspond to the water circulating in the soil macro-pores. About 30 mL of soil water samples were directly filtered in the field through a cellulose acetate filter at 0.2 μm (SARTORIUS) and used to determine the DOC concentrations, SUVA, Fe(II) following the protocol given by ANFOR^60^. Each filter was rinsed before use with ultrapure water and then with a few mL of the collected water. To determine the Fe_tot_ concentration and isotopic composition (δ^56^Fe), 1L of the water sample was collected under the inert (N_2_) atmosphere and stored in pre-cleaned, acid-washed polyethylene bottles, and then transported in the dark to the laboratory for filtration. This sample was filtered at 0.2 µm and acidified in an anaerobic chamber (JACOMEX) for a maximum of 4 h after sampling. The filtrates were stored in the dark at 4 °C before any analyses.

### Chemical analysis

The DOC concentration was determined using a total carbon analyzer (SHIMADZU TOC-V CSH, at Geosciences Rennes, France). The precision of the DOC measurements was estimated at ± 5% using a standard potassium hydrogen phthalate solution (SIGMA ALDRICH). Three-dimensional (3D) fluorescence spectra of the organic matter in samples at the beginning and end of each anoxic/oxic cycle were analyzed with a Perkin–Elmer LS 45 spectrofluorometer in a 10 mm quartz cuvette (Ecole Nationale Supérieure de Chimie de Rennes, France). The fluorescence spectra were treated with the PROGMEEF software in Matlab language^61^. The raw absorbance of the sample at 254 nm was corrected by the molar absorptivity of Fe(III)^62^. HIX was calculated as the ratio of the total emission intensity (λ_Em_) in the 435–480 nm region divided by the total emission intensity in the 300–345 nm region for an excitation (λ_Ex_) at 254 nm^30^. BIX was calculated as the ratio of intensities at λ_Em_ 380 nm and 430 nm for a λ_Ex_ at 310 nm^31^. SUVA (L mg^−1^ m^−1^) corresponded to the absorbance at 254 nm divided by the DOC concentration of the sample^32^. Increasing of SUVA, HIX, and BIX indicates an increase in the aromaticity, humic character, and autochthonous biological origin of the OM sources, respectively^30–32^.

The Fe concentration was determined using an AGILENT 7700X inductively coupled plasma mass spectrometer (ICP-MS) at Geosciences Rennes (University of Rennes, France) with a precision of 3% and 5% for > and < 1.8 µmol L^−1^ of Fe, respectively. The analytical interferences were eliminated and the digestion procedure was performed following Lotfi-Kalahroodi et al.^18^. The Fe(II) concentration was determined using the 1.10-phenanthroline colorimetric method^60^ at 510 nm using a UV–visible spectrometer (UV/VIS Spectrometer “Lambda 25” from PERKIN ELMER).

### Modelling calculations

Speciation calculation were performed using PHREEQC/Model VI^63^ specific to cation binding by organic matter. The “minteq.v4” database was completed by both (1) solubility constants of reduced soil phases eventually encountered, such as green rusts and hydroxyl-green rust^64^, and (2) specific binding parameters of Model VI corresponding to the complexation of Ca, Mg, Al(III), Fe(II) and Fe(III) with the organic matter. The specific binding parameters for Ca, Mg are from Tipping ^65^, those for Al(III) and Fe(III) from Marsac et al.^66^ and those for Fe(II) from Catrouillet et al.^34^.

### Iron isotope measurements and data calculations

Sample digestion and iron purification were performed following the protocol detailed in Lotfi-Kalahroodi et al.^18^, based on Rouxel et al.^67^. To briefly summarize, the acidified samples were digested in three steps using a mixture of (1) 22.6 mol L^−1^ HF and 14.6 mol L^−1^ HNO_3_, then (2) 12 mol L^−1^ HCl and 14.6 mol L^−1^ HNO_3_ and finally (3) suprapur 30% H_2_O_2_ and 14.6 mol L^−1^ HNO_3_. At each step, the sample was evaporated and dried. Iron was purified through anion exchange resin Dowex 1X8, chloride form (100–200 mesh). The residue of the purified Fe was evaporated to dryness and then dissolved in 0.28 mol L^−1^ of HNO_3_ for mass spectrometry analysis using a Thermo Neptune-plus multicollector inductively coupled plasma mass spectrometer (MC–ICP–MS) in medium mass resolution (IFREMER, Brest, France). The cups were set up to measure ^52^Cr, ^54^Fe, ^56^Fe, ^57^Fe, ^58^Fe, ^60^Ni, ^61^Ni, and ^62^Ni to correct ^54^Cr interference on ^54^Fe using ^52^Cr abundances. The ^62^Ni/^60^Ni ratio measurement allowed to correct the instrumental mass bias. Moreover, the IRMM-14 international reference material and a SPEX internal standard solution were used as sample-standard bracketing for each analytical run to determine the precision of the δ^56^Fe analysis. The average external precisions were found at 0.08‰ for δ^56^Fe of IRMM-14 (at 2 standard deviations, 2SD). A Ni reference solution was added to the standards and samples with a Ni:Fe ratio of 1:1. In general, the δ^56^Fe were measured at a concentration ranging from 18.0 to 1.8 µmol L^−1^ with a maximum difference of less than 10% between the concentrations of the samples and the standard.

The iron isotope composition of an unknown sample was reported as δ^56^Fe in per mil notation relative to IRMM-14 external standard, expressed as:

1 $$  \delta ^{{56}} Fe\left( \permil \right) = \left( {\frac{{\left( {{\raise0.7ex\hbox{${^{{56}} Fe}$} \!\mathord{\left/ {\vphantom {{^{{56}} Fe} {^{{54}} Fe}}}\right.\kern-\nulldelimiterspace} \!\lower0.7ex\hbox{${^{{54}} Fe}$}}} \right)_{{sample}} }}{{\left( {{\raise0.7ex\hbox{${^{{56}} Fe}$} \!\mathord{\left/ {\vphantom {{^{{56}} Fe} {^{{54}} Fe}}}\right.\kern-\nulldelimiterspace} \!\lower0.7ex\hbox{${^{{54}} Fe}$}}} \right)_{{IRMM - 014}} }} - 1} \right) \times 1000  $$

Since the initial studied soil in this experiment fractionated the δ^56^Fe values relative to IRMM-14 (about 0.43 ± 0.08 ‰), we introduce another notation (δ^56^Fe′), to compare δ^56^Fe of the soil solution with that of the initial soil. It corresponds to the Fe isotope composition of the filtered sample (δ^56^Fe_solution_) corrected from the soil Fe isotope composition (δ^56^Fe_soil_), such as:

2 $$ \delta ^{{56}}Fe_{{{\text{Solution}} - {\text{Soil}}}}^{{\prime }} \left( \textperthousand \right) = \delta ^{{56}}Fe_{{{\text{Solution}}}} - \delta ^{{56}}Fe_{Soil} $$

Procedural blanks, including the evaporation/digestion and ion exchange purification steps, were performed for each sample purification series. On average, these blanks contained 0.02 ± 0.01 nmol of Fe. Compared to the typical amount of Fe processed through the entire purification steps within the range of 8.4 nmol–1.61 µmol, the blanks represented less than 0.2% of Fe and were therefore negligible. An internal standard BHVO-1 (a Hawaiian basalt) with an average δ^56^Fe of 0.12 ± 0.08‰ (2SD, n = 32)^67^ was used to evaluate the accuracy of the method. Ferrozine assay was used to verify the yields of the sample purification steps^68,69^. In all cases, the Fe concentrations were below the detection limit, suggesting that less than 1% of Fe was lost during the purification (i.e. recovery > 99%).

For the samples i that were analyzed two times, the mean values of the duplicate analyses are reported with their 95% confidence interval (Supplementary Table S2). The error propagation (2SD) of sample i was reported with the calculation of maximum value between twofold standard deviation of average value of the two measured δ^56^Fe and a minimum value of their 2SD. If sample i was measured n times, the error propagation was calculated as:

3 $$ 2SD_{{\delta^{56} Fe_{i} }} = max\left( {2 SD \left( {\delta^{56} Fe_{{i_{1} }} , \ldots ,\delta^{56} Fe_{{i_{n} }} } \right) ,\min \left( {2SD_{{\delta^{56} Fe_{{i_{1} }} }} , \ldots ,2SD_{{\delta^{56} Fe_{{i_{n} }} }} } \right)} \right) $$

The δ^56^Fe of the size fraction n (δ^56^Fe_n,_ n: the total fraction, the < 3 µm fraction or the < 0.2 µmfraction), is calculated based on mass balance using:

4 $$ \delta ^{{56}}Fe_{n} = \sum \left( {\delta^{56} Fe_{i} \times \frac{{x_{i } }}{{\sum x_{i} }}} \right) $$

where x_i_ is the Fe amount in the fraction i. For example, δ^56^Fe_0.2 µm–30 kDa_ (e.g. n: < 0.2 µm, and i ≤ 30 kDa and 0.2 µm–30 kDa) can be calculated from Eq. (4) as follows:

5 $$ \delta^{56} Fe_{{0.2\upmu {\text{m}} - 30{\text{kDa}}}} = {{\left( {\delta^{56} Fe_{{ < 0.2\upmu {\text{m}}}} - \frac{{x_{{ < 30{\text{kDa}} }} }}{{x_{{ < 0.2\upmu {\text{m}}}} }}\delta^{56} Fe_{{B < 30{\text{kDa}} }} } \right)} \mathord{\left/ {\vphantom {{\left( {\delta^{56} Fe_{{ < 0.2\upmu {\text{m}}}} - \frac{{x_{{ < 30{\text{kDa}} }} }}{{x_{{ < 0.2\upmu {\text{m}}}} }}\delta^{56} Fe_{{B < 30{\text{kDa}} }} } \right)} {\left( {\frac{{x_{{0.2\upmu {\text{m}} - 30{\text{kDa}}}} }}{{x_{{ < 0.2\upmu {\text{m}}}} }}} \right)}}} \right. \kern-\nulldelimiterspace} {\left( {\frac{{x_{{0.2\upmu {\text{m}} - 30{\text{kDa}}}} }}{{x_{{ < 0.2\upmu {\text{m}}}} }}} \right)}} $$

The Fe isotopic composition and error propagations were calculated using a Monte Carlo simulation^18,70^ for the calculated size fractions (i.e. > 3 µm, 3–0.2 µm, and 0.2 µm–30 kDa fractions). The apparent difference in Fe isotope composition (Δ^56^Fe_A–B_) between various fractions (Supplementary Table S3) was calculated as:

6 $$ \Delta {}^{56}Fe_{{\left( {A - B} \right)}} \left( \textperthousand \right) = \delta {}^{56}Fe_{A} - \delta {}^{56}Fe_{B} $$

The twofold standard deviation (2SD) of the isotopic difference calculated between the various size fractions (Δ^56^Fe_3µm–0.2 µm_, and Δ^56^Fe_0.2 µm–30 kDa_) was calculated as:

7 $$ 2SD_{{\Delta^{56} Fe_{i - n} }} = \sqrt {\mathop \sum \limits_{i}^{n} (2SD_{i} )^{2} } $$

Besides, the 2SD of the δ^56^Fe′ (Eq. (2)) was calculated following Eq. (7).

## Supplementary Information

Supplementary Information.

## Data Availability

All data are available in this manuscript and the Supplementary information file.
