# Supplementary material for: More than redox, biological organic ligands control iron isotope fractionation in the riparian wetland
Source: Sci Rep. 2021 Jan 21;11:1933. doi: 10.1038/s41598-021-81494-z (PMC7820352; doi:10.1038/s41598-021-81494-z)
Supplement: Supplementary file 1 — Supplementary Information. [file 41598_2021_81494_MOESM1_ESM.pdf]

Supplementary information

More than redox, biological organic ligands control iron isotope fractionation in the riparian wetland

Elaheh Lotfi-Kalahroodi[1,*], Anne-Catherine Pierson-Wickmann[1], Olivier Rouxel[2], Remi Marsac[1], Martine LeCoz-Bouhnik[1], Khalil Hanna[3] and Mélanie Davranche[1]

[1] Univ. Rennes, CNRS, Géosciences Rennes - UMR 6118, 35000 Rennes, France

[2] IFREMER, Unité de Géosciences Marines, 29280 Plouzané, France

[3] Univ. Rennes, Ecole Nationale Supérieure de Chimie de Rennes, CNRS ISCR UMR6226, 35000 Rennes, France

Rayleigh distillation and equilibrium fractionation models during anoxic periods

In both anoxic periods 1 and 3, the 0.2 µm-30 kDa fractions had the highest $\delta^{56}Fe$ at 0.83 ± 0.14‰ and 0.72 ± 0.16‰ whereas, the soluble <30 kDa fractions exhibited negative $\delta^{56}Fe$ at -0.59 ± 0.09‰ and -0.59 ± 0.10‰, respectively. For the anoxic period 1, the Fe isotope fractionation between the > 3 µm, 3-0.2 µm and 0.2 µm-30 kDa fractions and the <30 kDa fraction were calculated at $\Delta^{56}Fe_{(>3\mu m)-(<30kDa)}$ = 1.01 ± 0.13‰, $\Delta^{56}Fe_{(3\text{-}0.2\ \mu m)-(<30\ kDa)}$ = 1.33 ± 0.28‰ and $\Delta^{56}Fe_{(0.2\ \mu m\text{-}30\ kDa)-(<30\ kDa)}$ = 1.41 ± 0.17‰, respectively (Table S3). Similarly, for the anoxic period 3, the Fe isotope fractionation between the > 3 µm, 3-0.2 µm and 0.2 µm-30 kDa fractions and the <30 kDa fraction were $\Delta^{56}Fe_{(>3\ \mu m)-(<30\ kDa)}$ = 1.02 ± 0.17‰, $\Delta^{56}Fe_{(3\text{-}0.2\ \mu m)-(<30\ kDa)}$ = 1.10 ± 0.71‰ and $\Delta^{56}Fe_{(0.2\ \mu m\text{-}30\ kDa)-(<30\ kDa)}$ = 1.31 ± 0.19‰, respectively. The production of such isotopically heavy Fe pool, both in particles and colloidal fraction so revealed the release of isotopically light Fe in the < 30 kDa fractions. Considering the enrichment of the > 30 kDa in heavy Fe isotopes due to DIR, simple isotopic mass balance suggests after about 62 to 83% (Fe(II)/Fe_{tot} in the 0.2µm-30kDa fractions; Table 1) of Fe(III) reduction through DIR, an isotopically heavy Fe pool of about 0.9 to 0.5‰ (Fractionation factor= 1.0012‰) or 1.8 to 0.8‰ (Fractionation factor= 1.003 ‰) should remain at the end of anoxic periods (Figs. S2 and S3). As previous studies demonstrated that dissimilatory Fe reduction can produce isotopically light Fe(II), with an isotopic fractionation varying -3< α < -1.2 ‰[1–4]. These fractionation factors were used in Rayleigh (open system) and equilibrium (closed system) fractionation systems to compare our results with these two models in Figures S2 and S3.

Figure legends:

Fig. S1. Transmission electron microscopy micrographs of Fe nanoaggregates embedded in an organic matrix in the 3-0.2 µm fractions for oxic periods a) 1 b) 2 and c) 3. Their sizes seemed to decrease with the redox cycles. Micrographs were performed after the deposition of 10 µL of each sample diluted in 1 mL of ethanol onto a 300-mesh copper grid coated with a lacey carbon film (Oxford Instruments, S166–3). The grids were observed using a transmission electron microscope (JEOL 2100 LAB6 operating at 200 kV) (THEMIS Analytical Facility at the University of Rennes 1).

Fig. S2. Evolution of $\delta^{56}Fe$ of phase A (Fe(II)) and phase B (Fe(III)) vs. f_A ($Fe(II)/Fe_{tot}$) fraction in closed equilibrium (straight lines) and Rayleigh (dashed lines) models during anoxic period 1, using an isotopic fractionation at -1.2‰.

Fig. S3. Evolution of $\delta^{56}Fe$ of phase A (Fe(II)) and phase B (Fe(III)) vs. f_A ($Fe(II)/Fe_{tot}$) fraction in closed equilibrium (straight lines) and Rayleigh (dashed lines) models during anoxic period 1, using an isotopic fractionation at -3.0 ‰.

Fig. S4. Evolution of $\delta^{56}Fe$ of phase A (Fe(II)) and phase B (Fe(III)) vs. f_A ($Fe(II)/Fe_{tot}$) fraction in closed equilibrium (straight lines) and Rayleigh (dashed lines) models during oxic period 1, using an isotopic fractionation at 2.9 ‰.

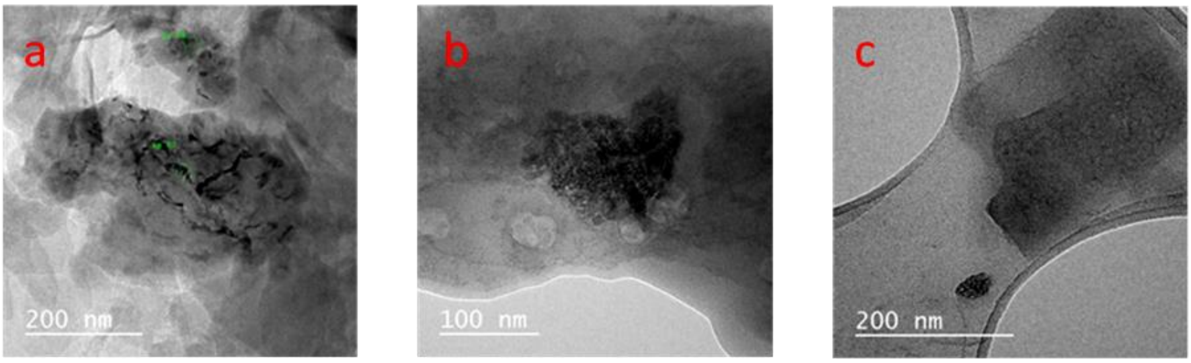

Fig. S1

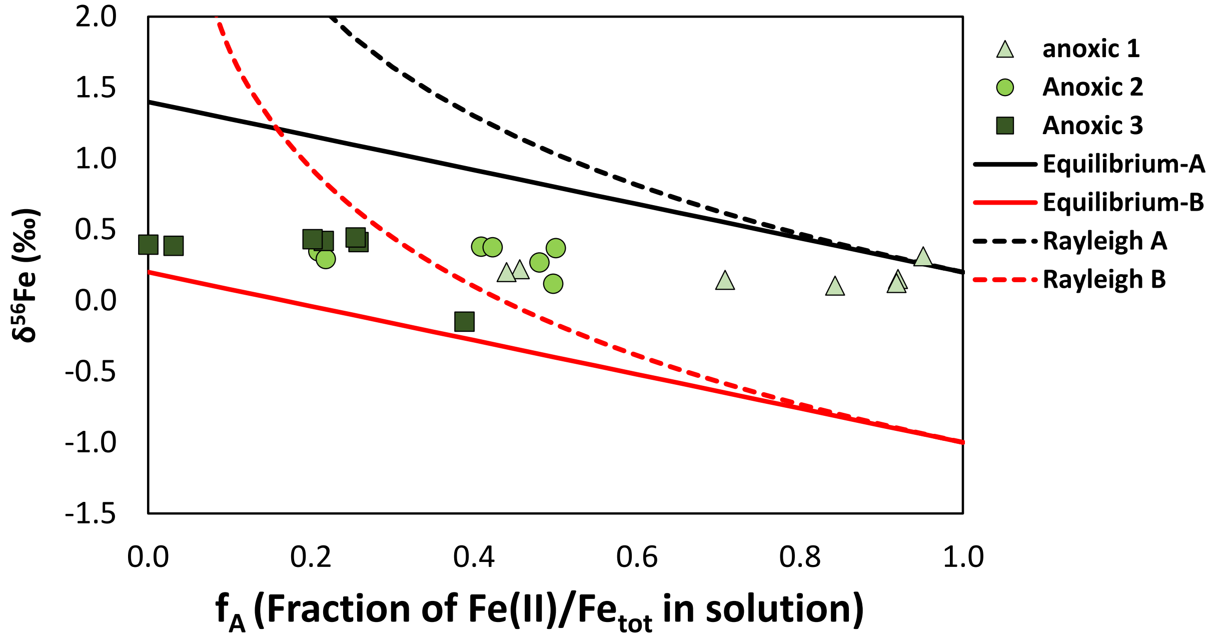
2.0
1.5
1.0
0.5
0.0
-0.5
-1.0
-1.5
0.0
0.2
0.4
0.6
0.8
1.0
δ^{56}Fe (‰)
f_A (Fraction of Fe(II)/Fe_{tot} in solution)
anoxic 1
Anoxic 2
Anoxic 3
Equilibrium-A
Equilibrium-B
Rayleigh A
Rayleigh B

Fig. S2

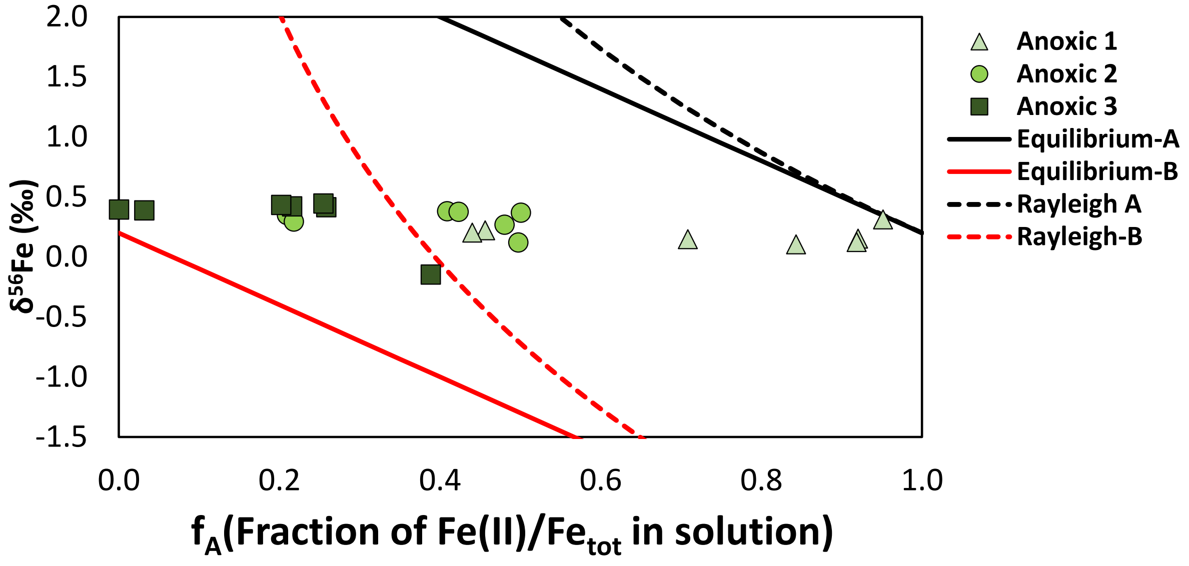
2.0
1.5
1.0
0.5
0.0
-0.5
-1.0
-1.5
δ56Fe (‰)
0.0
0.2
0.4
0.6
0.8
1.0
fA(Fraction of Fe(II)/Fetot in solution)
Anoxic 1
Anoxic 2
Anoxic 3
Equilibrium-A
Equilibrium-B
Rayleigh A
Rayleigh-B

Fig. S3

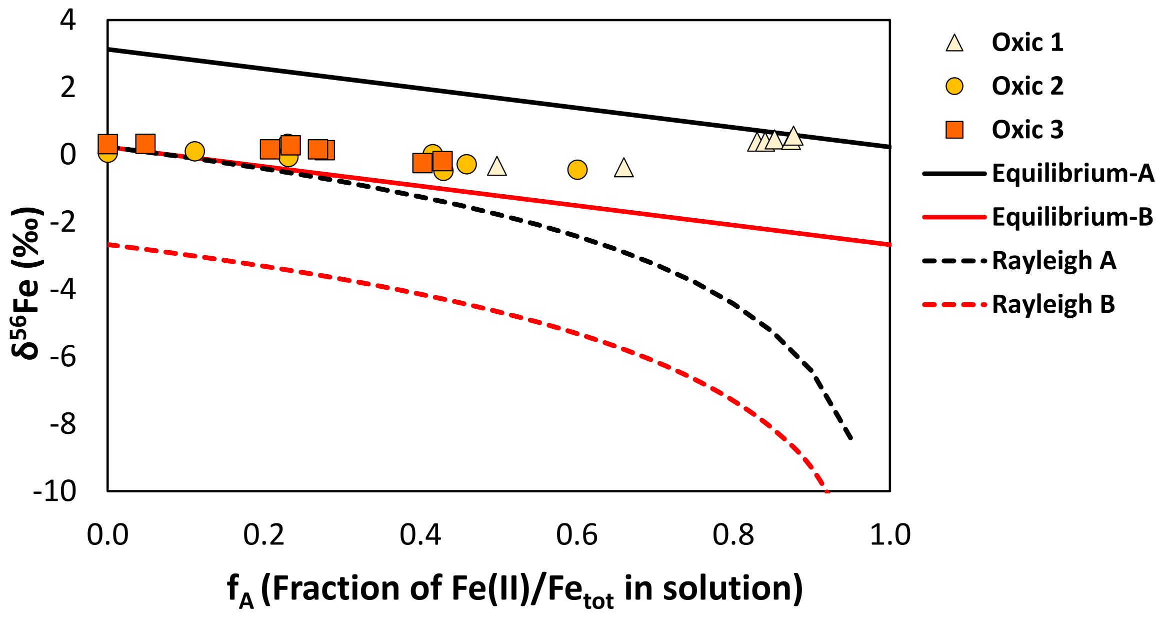

4
2
0
-2
-4
-6
-8
-10
δ56Fe (‰)
0.0
0.2
0.4
0.6
0.8
1.0
fA (Fraction of Fe(II)/Fetot in solution)
Oxic 1
Oxic 2
Oxic 3
Equilibrium-A
Equilibrium-B
Rayleigh A
Rayleigh B

Fig. S4

Table S1 - Chemical and isotopic compositions of the soil solution (<0.2μm) in the anoxic/oxic cycles. The iron isotopic composition of the initial wetland soil was measured at 0.43 ± 0.08‰.

Time (day)	pH	Eh (mV)	DOC (mmol L^{-1})	Fe (II) (μmol L^{-1})	Fe (III)(μmol L^{-1})	Fe_{tot}(μmol L^{-1})	δ^{56}Fe±2SD (‰)	$\delta^{56}Fe'_{sample-soil}$ ±2SD (‰)	Fe(II)/Fe_{tot}
**Anoxic 1**									
1, 0h	5.6	426	0.7 ± 0.1	2.6 ± 0.1	11.3 ± 0.4	13.8 ± 0.4	0.31 ± 0.13	-0.11 ± 0.15	0.19
2	6.0	391	1.6 ± 0.1	6.7 ± 0.3	16.0 ± 0.8	22.6 ± 0.7	0.15 ± 0.13	-0.27 ± 0.15	0.29
3	6.2	361	1.5 ± 0.1	8.8 ± 0.4	14.4 ± 0.8	23.2 ± 0.7	0.12 ± 0.06	-0.30 ± 0.10	0.38
6	6.5	278	2.4 ± 0.1	23.8 ± 1.2	20.9 ± 1.8	44.7 ± 1.3	0.10 ± 0.06	-0.32 ± 0.10	0.53
11	7.0	251	3.2 ± 0.2	46.6 ± 2.3	36.4 ± 3.4	83.1 ± 2.5	0.15 ± 0.08	-0.28 ± 0.11	0.56
16	7.6	-63	11.8 ± 0.6	96.6 ± 4.8	58.3 ± 6.7	154.9 ± 4.6	0.22 ± 0.06	-0.21 ± 0.10	0.62
20	7.5	-83	15.1 ± 0.8	124.2 ± 6.2	35.4 ± 7.8	159.5 ± 4.8	0.20 ± 0.06	-0.23 ± 0.10	0.78
**Anoxic 2**									
38, 0h	7.0	218	3.1 ± 0.2	99.4 ± 5.0	43.8 ± 6.6	143.2 ± 4.3	0.12 ± 0.06	-0.31 ± 0.10	0.69
39	7.1	79	3.7 ± 0.2	113.6 ± 5.7	28.7 ± 7.1	142.3 ± 4.3	0.37 ± 0.06	-0.06 ± 0.10	0.80
40	7.0	-81	3.7 ± 0.2	116.3 ± 5.8	31.7 ± 7.3	148.0 ± 4.4	0.27 ± 0.06	-0.16 ± 0.10	0.79
43	7.1	-103	4.4 ± 0.2	123.6 ± 6.2	44.9 ± 8.0	168.5 ± 5.1	0.38 ± 0.06	-0.05 ± 0.10	0.73
48	7.2	-186	3.9 ± 0.2	128.8 ± 6.4	35.5 ± 7.5	164.4 ± 3.9	0.38 ± 0.06	-0.05 ± 0.10	0.78
54	7.1	-149	10.3 ± 0.5	188.0 ± 9.4	37.2 ± 11.6	225.3 ± 6.8	0.35 ± 0.09	-0.08 ± 0.12	0.83

57	7.1	-175	9.5 ± 0.5	184.8 ± 9.2	37.9 ± 11.4	222.7 ± 6.7	0.29 ± 0.09	-0.13 ± 0.12	0.83
**Anoxic 3**									
75, 0h	6.9	136	3.6 ± 0.2	182.9 ± 9.1	0.0 ± 10.5	174.3 ± 5.2	-0.15 ± 0.14	-0.58 ± 0.16	1.00
76	7.0	-16	3.8 ± 0.2	202.9 ± 10.1	20.5 ± 12.2	223.4 ± 6.7	0.42 ± 0.06	-0.01 ± 0.10	0.91
77	7.2	-49	3.9 ± 0.2	180.8 ± 9.0	30.4 ± 11.0	211.2 ± 6.3	0.41 ± 0.06	-0.01 ± 0.10	0.86
80	7.1	-73	4.5 ± 0.2	212.9 ± 10.6	0.0 ± 12.4	212.3 ± 6.4	0.45 ± 0.06	0.02 ± 0.10	1.00
85	7.1	-125	4.3 ± 0.2	217.9 ± 10.9	9.3 ± 12.9	227.3 ± 6.8	0.43 ± 0.06	0.01 ± 0.10	0.96
90	7.2	-127	9.3 ± 0.5	231.1 ± 11.6	53.7 ± 14.4	284.8 ± 8.5	0.39 ± 0.06	-0.03 ± 0.10	0.81
94	7.1	-175	9.7 ± 0.5	241.3 ± 12.1	34.5 ± 14.6	275.9 ± 8.3	0.39 ± 0.11	-0.04 ± 0.14	0.87
**Time (day)**	**pH**	**Eh (mV)**	**DOC (mmol L^{-1})**	**Fe (II) (µmol L^{-1})**	**Fe (III)(µmol L^{-1})**	**Fe_{tot}(µmol L^{-1})**	**δ^{56}Fe±2SD (‰)**	**$\delta^{56}Fe'_{sample-soil}$ ±2SD (‰)**	**Fe(II)/Fe_{tot}**
**Oxic 1**									
21, 0h	6.9	-72	3.9 ± 0.2	52.9 ± 2.6	71.4 ± 4.6	124.3 ± 3.7	0.23 ± 0.13	-0.20 ± 0.15	0.43
21, 1h	6.7	45	3.3 ± 0.2	47.1 ± 2.4	33.0 ± 3.4	80.1 ± 2.4	-0.33 ± 0.09	-0.76 ± 0.12	0.59
21, 2h	6.6	142	3.5 ± 0.2	13.5 ± 0.7	40.7 ± 1.8	54.3 ± 1.6	-0.37 ± 0.07	-0.80 ± 0.11	0.25
21, 10h	6.3	198	2.2 ± 0.1	5.9 ± 0.3	21.1 ± 0.9	27.1 ± 0.8	0.38 ± 0.06	-0.05 ± 0.10	0.22
22	6.3	252	1.7 ± 0.1	4.7 ± 0.2	20.7 ± 0.8	25.4 ± 0.8	0.38 ± 0.06	-0.05 ± 0.10	0.19
25	6.4	550	2.5 ± 0.1	10.3 ± 0.5	13.3 ± 0.9	23.5 ± 0.7	0.45 ± 0.06	0.02 ± 0.10	0.44
31	6.5	532	2.1 ± 0.1	6.9 ± 0.3	13.3 ± 0.7	20.2 ± 0.6	0.43 ± 0.06	0.00 ± 0.10	0.34

37	6.4	541	2.2 ± 0.1	6.1 ± 0.3	13.5 ± 0.7	19.6 ± 0.6	0.56 ± 0.06	0.13 ± 0.10	0.31
**Oxic 2**									
58, 0h	6.9	-140	3.2 ± 0.2	161.2 ± 8.1	23.4 ± 9.8	184.6 ± 5.5	0.32 ± 0.09	-0.11 ± 0.12	0.87
58, 1h	6.8	74	3.5 ± 0.2	145.6 ± 7.3	38.8 ± 9.1	184.5 ± 5.5	-0.07 ± 0.06	-0.49 ± 0.10	0.79
58, 2h	6.7	147	4.4 ± 0.2	110.8 ± 5.5	26.1 ± 6.9	136.9 ± 4.1	-0.48 ± 0.06	-0.91 ± 0.10	0.81
58, 10h	6.8	121	4.2 ± 0.2	61.3 ± 3.1	34.5 ± 4.2	95.8 ± 2.9	-0.44 ± 0.06	-0.87 ± 0.10	0.64
59	6.7	197	3.1 ± 0.2	111.8 ± 5.6	18.0 ± 6.8	129.8 ± 3.9	-0.28 ± 0.06	-0.71 ± 0.10	0.86
62	7.0	226	4.4 ± 0.2	125.3 ± 6.3	114.5 ± 9.5	239.8 ± 7.2	0.06 ± 0.09	-0.37 ± 0.12	0.52
68	6.9	141	2.5 ± 0.1	92.1 ± 4.6	121.0 ± 7.9	213.1 ± 6.4	0.10 ± 0.10	-0.33 ± 0.13	0.43
74	6.7	147	3.6 ± 0.2	117.0 ± 5.9	23.2 ± 8.7	140.2 ± 6.4	0.02 ± 0.06	-0.41 ± 0.10	0.83
**Oxic 3**									
95, 0h	6.8	-118	3.6 ± 0.2	183.6 ± 9.2	24.5 ± 11.1	208.1 ± 6.2	0.28 ± 0.16	-0.14 ± 0.18	0.88
95, 1h	6.8	7	4.0 ± 0.2	142.2 ± 7.1	73.2 ± 9.6	215.4 ± 6.5	0.16 ± 0.06	-0.27 ± 0.10	0.66
95, 2h	7.1	-3	4.8 ± 0.2	142.8 ± 7.1	53.5 ± 9.3	196.3 ± 5.9	0.14 ± 0.06	-0.29 ± 0.10	0.73
95, 10h	7.0	91	3.9 ± 0.2	140.8 ± 7.0	14.6 ± 8.4	155.4 ± 4.7	-0.19 ± 0.14	-0.61 ± 0.17	0.91
96	6.6	121	2.9 ± 0.1	141.8 ± 7.1	20.6 ± 8.6	162.4 ± 4.9	-0.25 ± 0.06	-0.68 ± 0.10	0.87
99	7.1	66	3.3 ± 0.2	185.0 ± 9.2	13.7 ± 11.0	198.7 ± 6.0	0.16 ± 0.06	-0.27 ± 0.10	0.93
105	7.0	-80	4.7 ± 0.2	193.3 ± 9.7	65.3 ± 12.4	258.6 ± 7.8	0.32 ± 0.06	-0.11 ± 0.10	0.75

111	7.0	-97	4.2 ± 0.2	260.0 ± 13.0	11.7 ± 15.3	271.7 ± 8.2*	0.31 ± 0.10	-0.12 ± 0.13	0.96
111**	6.1	571	2.7 ± 0.1	1.3 ± 0.1	10.2 ± 0.5	11.5 ± 0.6	0.52 ± 0.10	0.09 ± 0.13	0.11

* DOC: Dissolved organic carbon.

** After total oxidation: measured parameters after that rubber stopper of reactor has been opened.

Table S2 – The Fe isotopic compositions of the soil suspension and fluorescence indexes of the soil solution. HIX = humification index[5]; BIX = biological index[6]; SUVA = specific UV absorbance[7].

Period	Time (day)	δ^{56}Fe ± 2SD (‰) Soil suspension	SUVA $_{\lambda=254\ nm}$ (L mg^{-1} m^{-1}) soil solution	HIX soil solution	BIX soil solution
**Anoxic-1**	1	nd	3.14	3	0.36
	20	0.62 ± 0.09	0.20	9	0.42
**Oxic-1**	21	0.51 ± 0.09	1.82	19	0.40
	37	0.50 ± 0.09	0.98	7	0.38
**Anoxic-2**	38	0.54 ± 0.09	5.38	44	0.35
	57	0.47 ± 0.09	2.43	31	0.40
**Oxic2**	58	0.52 ± 0.09	4.56	36	0.36
	74	0.58 ± 0.13	4.02	31	0.39
**Anoxic-3**	75	0.51 ± 0.13	4.57	61	0.38
	94	0.61 ± 0.13	2.37	22	0.42
**Oxic-3**	95	0.55 ± 0.13	6.65	32	0.38
	111	0.56 ± 0.13	3.45	16	0.44

The errors of δ^{56}Fe ware calculated through standard deviation (2SD) of standard bracketing.

Table S3- Iron isotopic fractionation ($\Delta^{56}Fe_{A-B}$) between the size fraction A (on the left, yellow column) and the size fraction B (on the top, blue row) for anoxic periods 1 and 3 and oxic period 1 and 3. The values were calculated by subtracting the δ^{56}Fe of the fraction B from the δ^{56}Fe of the fraction A.

**$\Delta^{56}Fe_{A-B}$**	**Size fraction B**		
**Size fraction A**	**3-0.2 µm**	**0.2µm-30 kDa**	**<30 kDa**
**Anoxic 1**			
**>3 µm**	-0.32 ± 0.28	-0.40 ± 0.17	1.01 ± 0.13
**3-0.2 µm**	--	-0.08 ± 0.30	1.33 ± 0.28
**0.2µm-30 kDa**	0.08 ± 0.30	--	1.41 ± 0.17
**Anoxic 3**			
**>3 µm**	-0.08 ± 0.71	-0.29 ± 0.21	1.02 ± 0.17
**3-0.2 µm**	--	-0.22 ± 0.72	1.10 ± 0.71
**0.2µm-30 kDa**	0.22 ± 0.72	--	1.31 ± 0.19
**Oxic 1**			
**>3 µm**	0.13 ± 0.13	0.10 ± 0.26	-0.12 ± 0.10
**3-0.2 µm**	--	-0.02 ± 0.27	-0.25 ± 0.11
**0.2µm-30 kDa**	0.02 ± 0.27	--	ns
**Oxic 3**			
**>3 µm**	0.11 ± 0.17	0.21 ± 0.52	-0.02 ± 0.19
**3-0.2 µm**	--	0.09 ± 0.51	-0.14 ± 0.18
**0.2µm-30 kDa**	-0.09 ± 0.51	--	-0.23 ± 0.52

---: Not defined isotopic fractionation.

Table S4 - Chemical and isotopic compositions of the natural soil solution filtrate at 0.2 µm during seasonal anoxic and oxic periods in the wetland.

Date	DOC* (mmol L^{-1})	SUVA	Fe (II) (µmol L^{-1})	Fe_{tot} (µmol L^{-1})	$\delta^{56}Fe \pm 2SD$ (‰)
**Oxic**					
**20/01/2017**	1.0 ± 0.1	4.5	nd	60 ± 3	-0.21 ± 0.06
**09/02/2017**	1.9 ± 0.1	2.7	nd	15 ± 1	0.04 ± 0.14
**Anoxic**					
**21/02/2017**	10.8 ± 0.5	3.2	nd	382 ± 19	-0.54 ± 0.09
**08/03/2017**	11.3 ± 0.0	2.8	74 ± 4	509 ± 25	-0.46 ± 0.09
**21/03/2017**	10.8 ± 0.5	3.4	93 ± 5	475 ± 24	-0.38 ± 0.09
**04/04/2017**	6.7 ± 0.3	4.8	160 ± 8	413 ± 21	-0.24 ± 0.14
**02/05/2017**	1.6 ± 0.1	5.9	221 ± 11	645 ± 32	-0.80 ± 0.07
**Oxic**					
**01/06/2017**	nd	nd	78 ± 4	314 ± 16	nd

* DOC: Dissolved organic carbon. nd: not determined.

Table S5 – Chemical composition of the soil used to prepare soil solutions collected at 0-10cm depth. The analysis was performed by SARM (France).

Compound	SiO_2	Al_2O_3	Fe_2O_3	MnO	MgO	CaO	Na_2O	K_2O	TiO_2	P_2O_5	Total
(wt. %)	63.04	10.15	5.23	0.10	0.57	0.29	0.42	1.79	0.78	0.17	100.13

79

Workflow schema

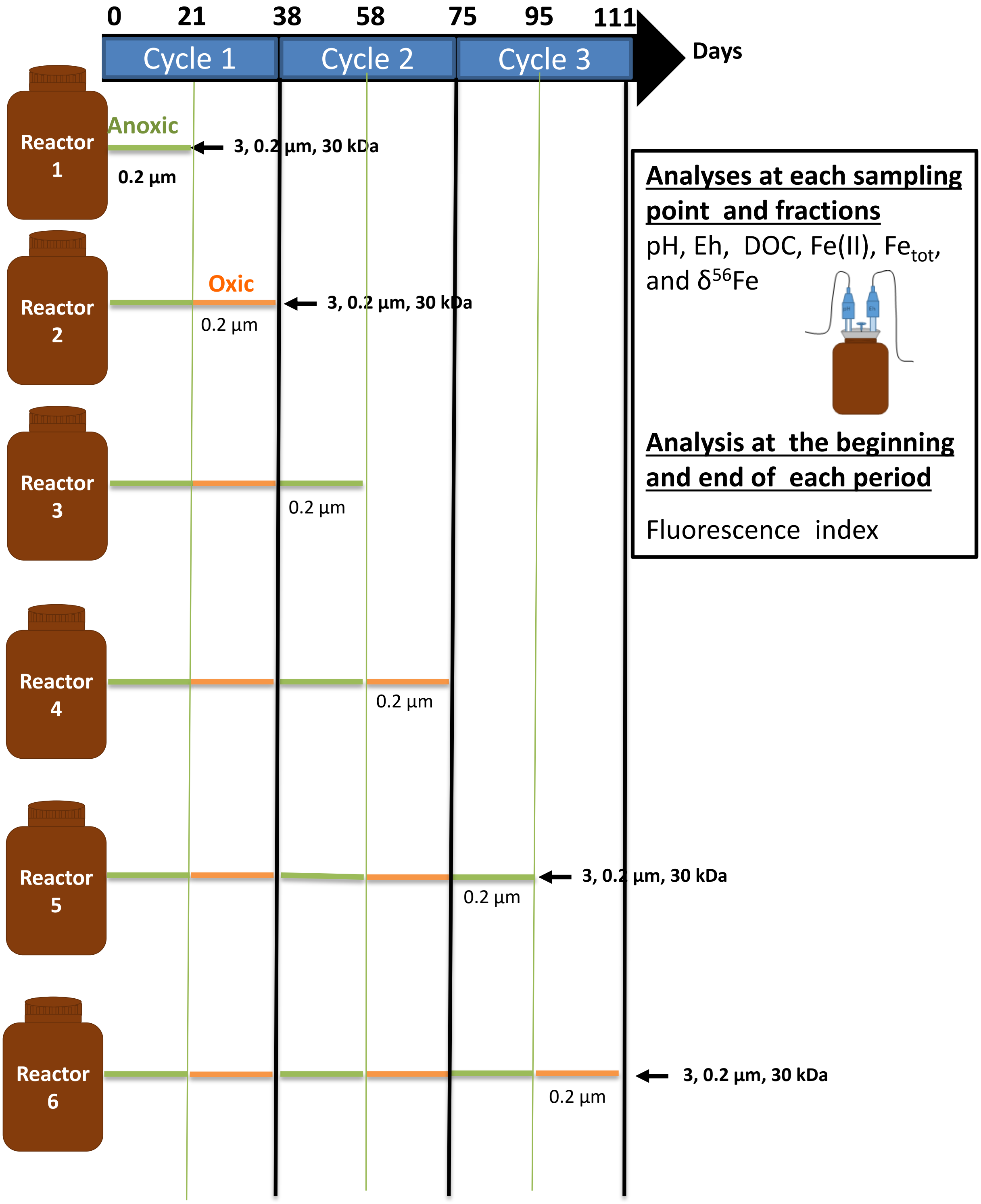

80

References

1. Crosby, H. A., Johnson, C. M., Roden, E. E. & Beard, B. L. Coupled Fe (II)− Fe (III) electron and atom exchange as a mechanism for Fe isotope fractionation during dissimilatory iron oxide reduction. *Environ. Sci. Technol.* **39**, 6698–6704 (2005).
2. Icopini, G. A., Anbar, A. D., Ruebush, S. S., Tien, M. & Brantley, S. L. Iron isotope fractionation during microbial reduction of iron: the importance of adsorption. *Geology* **32**, 205–208 (2004).
3. Crosby, H. A., Roden, E. E., Johnson, C. M. & Beard, B. L. The mechanisms of iron isotope fractionation produced during dissimilatory Fe(III) reduction by Shewanella putrefaciens and Geobacter sulfurreducens. *Geobiology* **5**, 169–189 (2007).
4. Johnson, C. M., Roden, E. E., Welch, S. A. & Beard, B. L. Experimental constraints on Fe isotope fractionation during magnetite and Fe carbonate formation coupled to dissimilatory hydrous ferric oxide reduction. *Geochim. Cosmochim. Acta* **69**, 963–993 (2005).
5. Zsolnay, A., Baigar, E., Jimenez, M., Steinweg, B. & Saccomandi, F. Differentiating with fluorescence spectroscopy the sources of dissolved organic matter in soils subjected to drying. *Chemosphere* **38**, 45–50 (1999).
6. Huguet, A. *et al.* Properties of fluorescent dissolved organic matter in the Gironde Estuary. *Org. Geochem.* **40**, 706–719 (2009).
7. Matilainen, A. *et al.* An overview of the methods used in the characterisation of natural organic matter (NOM) in relation to drinking water treatment. *Chemosphere* **83**, 1431–1442 (2011).
